# Supplementary material for: Knowledge, Attitudes, and Perception towards COVID-19 Vaccination among the Adult Population: A Cross-Sectional Study in Turkey
Source: Vaccines (Basel). 2022 Feb 11;10(2):278. doi: 10.3390/vaccines10020278 (PMC8877322; doi:10.3390/vaccines10020278)
Supplement: Supplementary file 1 [file vaccines-10-00278-s001.zip › vaccines-1588735-supplementary.pdf]

Questionnaire form

Contact information	
If you have any concerns, questions, and complaints about this study, its risks, benefits, and procedures contact the Protocol Director, Dr. Meliha Cagla Sonmezer at (+90) 505 892 32 78. *Independent Contact:* If you are not satisfied with how this study is being conducted, or if you have any concerns, complaints, or general questions about the research or your rights as a participant, please contact the Hacettepe University Institutional Review Board to speak to someone independent of the research team at (+90) 312-305-1082 or email at goetik@hacettepe.edu.tr. If you agree to participate in this research, please select "Yes" below.	
**Informed consent**	
We have conducted a survey entitled "Knowledge, Attitudes and Perception towards COVID-19 Vaccination among Adult Population: A Cross-Sectional Study in Turkey". The results of the study will represent the knowledge, attitudes and perceptions towards COVID-19 vaccinations. The survey may take 10-15 minutes. Nowhere in the survey, you won't be asked for your personal information. All of your information will be kept secret. You have the right to participate or deny, and during the time of participation, you can withdraw yourself from responding. The study will not be benefitted you by money or other compensations but the outcome of the study may consider by the policymakers and take initiative for COVID-19 vaccinations in Turkey. Thank you for your participation.	
Are you willing to participate in the survey?	o Yes<br>o No
**Section 1: Sociodemographic data**	
1. Age	………………………..
2. Gender	o Female<br>o Male
3. Chronic Disease	o No<br>o Hypertension<br>o Asthma/COPD<br>o Diabetes<br>o Chronic Heart Disease<br>o Cancer<br>o Chronic Kidney Disease<br>o Other
4. Use of regular medications	o Yes<br>o No
5. Educational Level	o No education<br>o Primary school<br>o Secondary school<br>o Bachelor degree<br>o Higher education
6. Occupational status	o Employed<br>o Non-employed<br>o Retired
7. Influenza shot in the past years	o Yes<br>o No

8. Having a child	o Yes<br>o No
9. My child received all the recommended vaccines	o Yes<br>o No
10. Have you been diagnosed with COVID-19?	o Yes<br>o No
11. Which of the following sources will you choose to derive Covid-19 vaccine-related information? (multiple choices)	o Recommendation from health authorities<br>o Social media<br>o Friends<br>o Recommendation from my relatives who are healthcare workers<br>o Other sources
**Section 2: Knowledge, Attitude and Perception**	
1. Do you believe that the vaccines will produce immune response against COVID-19 ?	o Yes<br>o No
2. Everyone should get vaccinated to end the COVID-19 pandemic.	o Disagree<br>o Neither agree or disagree<br>o Agree
3. Although we are not able to vaccinate everybody, the pandemic will end soon.	o Disagree<br>o Neither agree or disagree<br>o Agree
4. People who have had COVID-19 and recovered no need to get vaccinated.	o Disagree<br>o Neither agree or disagree<br>o Agree
5. The vaccine's protection is achieved immediately after getting the COVID-19 vaccine.	o Disagree<br>o Neither agree or disagree<br>o Agree
6.Stop practicing precautions such as masking, social distancing, and hand hygiene after receiving COVID-19 vaccine.	o Disagree<br>o Neither agree or disagree<br>o Agree
7. COVID-19 pandemic changed my approach to vaccination.	o Disagree<br>o Undecided<br>o Agree
8. Worried about family members becoming ill with COVID-19.	o Disagree<br>o Undecided<br>o Agree
